# Real-world effectiveness of molecular-matched therapies in salivary gland cancer

**DOI:** 10.1016/j.esmoop.2026.106961

**Published:** 2026-04-30

**Authors:** J.A.M. Weijers, N.J. van Ruitenbeek, A.C.H. van Engen-van Grunsven, C.M.L. Driessen, L.A. Devriese, M. Slingerland, A. Hoeben, S.F. Oosting, W.H. Schreuder, A. Sewnaik, S. van Helvert, J.A. Schalken, G.W. Verhaegh, C.M.L. van Herpen

**Affiliations:** 1 Department of Medical Oncology, Radboud Institute for Medical Innovation, Radboud University Medical Center, Nijmegen, Netherlands; 2 Department of Pathology, Radboud Institute for Medical Innovation, Radboud University Medical Center, Nijmegen, Netherlands; 3 Department of Medical Oncology, University Medical Centre Utrecht, Utrecht, Netherlands; 4 Department of Medical Oncology, Leiden University Medical Centre, Leiden, Netherlands; 5 Division of Medical Oncology, Department of Internal Medicine, Maastricht UMC+ Comprehensive Cancer Centre, GROW-School of Oncology and Reproduction, Maastricht University Medical Centre+, Maastricht, Netherlands; 6 Department of Medical Oncology, University Medical Centre Groningen, University of Groningen, Groningen, Netherlands; 7 Department of Head and Neck Surgery & Oncology, The Netherlands Cancer Institute, Amsterdam, Netherlands; 8 Department of Otorhinolaryngology, Head and Neck Surgery, Erasmus Medical Centre, Rotterdam, Netherlands; 9 Department of Urology, Radboud Institute for Medical Innovation, Radboud University Medical Center, Nijmegen, Netherlands

**Keywords:** adenoid cystic carcinoma, real-world data, salivary duct carcinoma, systemic therapy, targeted therapy, salivary gland cancer

## Abstract

**Background:**

Salivary gland cancer (SGC) is a rare cancer comprising over 20 subtypes. Although its molecular landscape is increasingly characterised, real-world data on molecular-matched therapies (MMTs) remain limited, and predictive biomarkers are needed.

**Patients and methods:**

Since 2017, real-world data of patients with SGC attending the outpatient clinic at Radboud university medical center have been systematically collected. Best overall response to MMT was assessed per RECIST v1.1. Median progression-free survival (mPFS) was estimated using Kaplan–Meier statistics and compared between the molecular subgroups using the log-rank test.

**Results:**

At data cutoff, the database contained 662 patients with SGC, including 464 patients with recurrent and/or metastatic disease. Among them, 381 patients exhibited ≥1 molecular alteration, of whom 54% received MMT. Among patients with salivary duct carcinoma (SDC), 83% of patients with ≥1 molecular alteration received MMT, compared with 25% with adenoid cystic carcinoma. In 110 patients with SDC, mPFS with any first-line androgen receptor axis–targeted therapy was 5.3 months [95% confidence interval (CI) 3.5-7.2 months] and was significantly longer in *HRAS*-mutant (19.1 months; 95% CI 11.0-27.2 months; *n* = 18) versus *HRAS*–wild-type cases (3.8 months; 95% CI 2.0-5.6 months; *n* = 64; *P* = 0.007). With the first human epidermal growth factor receptor 2-targeted therapy given (*n* = 35), mPFS was 8.5 months (95% CI 6.4-10.6 months), with objective responses in 61% of RECIST-assessable patients (*n* = 28). Additionally, objective responses were achieved with other MMTs, including vemurafenib/cobimetinib in *BRAF* V600E-mutant SDC (*n* = 3), larotrectinib in secretory carcinoma with *ETV6*::*NTRK3* gene fusions (*n* = 2), and ipilimumab/nivolumab in mucoepidermoid carcinoma with high tumour mutational burden (*n* = 1).

**Conclusions:**

Comprehensive molecular testing in SGC may allow access to MMT, with a subset of patients experiencing clinical benefit from this strategy.

## Highlights

• In a real-world R/M salivary gland cancer cohort, 54% of patients with a molecular alteration received matched therapy.
• A subset of patients with salivary gland cancer demonstrated clinical benefit from molecular-matched therapies.
• The effectiveness of AR axis–targeted and HER2–targeted therapies in salivary duct carcinoma was confirmed in a real-world cohort.
• *HRAS* mutations may predict the effectiveness of AR axis–targeted therapy in salivary duct carcinoma.

## Introduction

Salivary gland cancer (SGC) is a rare cancer type, with global crude incidence and mortality rates of 0.69 and 0.29 cases per 100 000 people per year, respectively.^1^ The most recent World Health Organization classification of head and neck tumours distinguishes >20 histopathological subtypes, making each subtype even less common.^2^ The incidence of recurrent and/or metastatic (R/M) disease is highly variable among subtypes, with the highest rates in adenoid cystic carcinoma (AdCC, 55%), salivary duct carcinoma (SDC, 54%), myoepithelial carcinoma (42%), acinic cell carcinoma (29%), and secretory carcinoma (19%).^3^, ^4^, ^5^, ^6^

The rarity and heterogeneity of SGC hinder large-scale patient accrual in clinical trials, resulting in a lack of registered systemic therapies for most subtypes.^7^^,^^8^ Over the past decade, the molecular landscape of SGC has been increasingly characterised. In current routine practice, molecular markers are mainly applied for diagnosis and subtype classification.^9^ In addition, numerous actionable molecular targets have been identified.^10^^,^^11^ These targets could be leveraged to enable the rational application of molecular-matched therapies (MMTs) tailored to the individual patient’s tumour. Due to the low incidence of most SGC subtypes and molecular alterations, MMTs are often investigated within tumour-agnostic basket trials, including agents targeting the Notch signalling pathway, *NTRK* gene fusions, and human epidermal growth factor receptor 2 (HER2) overexpression.^12^, ^13^, ^14^, ^15^ Subtype-specific clinical trials have been limited to SDC and AdCC, in which MMTs targeting frequent molecular features have demonstrated efficacy.^16^, ^17^, ^18^

SDC has been the most widely explored SGC subtype with respect to MMTs. SDC frequently expresses the androgen receptor (AR) and/or HER2. As AR is expressed in 73%-100% of SDC cases, the AR signalling axis provides a targetable pathway for most patients.^4^^,^^19^^,^^20^ In a phase II trial, combined androgen blockade using a gonadotropin-releasing hormone analogue and the AR antagonist bicalutamide yielded an objective response rate (ORR) of 42%, median progression-free survival (mPFS) of 8.8 months, and median overall survival (mOS) of 30.5 months.^16^ HER2 status was positive in 29%-46% of SDC cases.^4^^,^^20^^,^^21^ In a phase II trial investigating trastuzumab/docetaxel in HER2-positive SDC, the ORR was 70.2%, with mPFS of 8.9 months and mOS of 39.7 months.^17^ More recently, the antibody-drug conjugate trastuzumab–deruxtecan demonstrated an ORR of 58.8% and mPFS of 20.5 months in two pooled phase I studies.^15^ In AdCC, the NOTCH inhibitors AL101 and brontictuzumab demonstrated clinical activity in patients with NOTCH-activated tumours, achieving partial response (PR) in 17% and stable disease (SD) in 55% of patients.^18^

Despite advances in MMTs for SGC in clinical trials, real-world evidence regarding their effectiveness remains limited. In addition, there is a clinical need for routinely available predictive biomarkers to guide treatment decisions in SDC. In this analysis of our real-world SGC cohort, we characterise the molecular landscape across subtypes, evaluate the clinical outcomes of MMTs, and explore predictive biomarkers for treatment effectiveness.

## Patients and methods

### Patients

A real-world database was set up to systematically collect data from patients with SGC at Radboud university medical center (Radboudumc), a tertiary SGC expertise centre in the Netherlands. At Radboudumc, all pathology diagnostics of patients with SGC are routinely reviewed by an expert pathologist at referral, with additional testing carried out as indicated. Since 2017, patients with SGC attending the outpatient clinics of the departments of Medical Oncology, Otorhinolaryngology, and Maxillofacial Surgery were invited to provide informed consent for participation in the Radboudumc Biobank Salivary Gland Cancers. This biobank facilitates clinical data acquisition and the archiving of tissue samples and has been approved by the institutional review board (file number 2017-3697 and 2019-5476). In addition to this biobank, patient data were also included retrospectively according to the ‘Consent at the Gate Procedure’ of Radboudumc. This procedure enables the inclusion of patients based on active permission, whereby patients aged 16 years and older are asked to consent to the use of their health data and remaining tissue samples for scientific research. This study was approved by the local medical ethics committee of the Radboudumc (2024-17358) and conducted according to the principles of the Declaration of Helsinki.

### Data collection

Data were manually extracted from electronic patient records and collected in the cloud-based data capture system Castor (www.castoredc.com). Data were included but were not limited to patient characteristics, symptoms, dates of diagnosis and R/M disease, sites of the primary tumour and metastases, pathological features, molecular tumour diagnostics, treatment, therapy responses, survival data, and follow-up information. In addition, molecular data obtained by Lassche et al.^11^ were incorporated into the database. MMTs were administered as reimbursed care, in clinical trials, or under compassionate use. A subset of the current cohort overlaps with previously published case series of AR axis–targeted therapy (hereafter referred to as AR–targeted therapy; *n* = 35)^22^ and HER2–targeted therapy (*n* = 13)^23^ for SDC, TRK inhibitors for secretory carcinoma (*n* = 1),^24^ and with several clinical trials.^25^, ^26^, ^27^, ^28^

### Molecular tumour diagnostics and MMT

At Radboudumc, DNA next-generation sequencing (NGS) and RNA gene fusion analysis based on anchored-multiplex PCR are being carried out as routine practice for patients with R/M SGC since 2016 and 2018, respectively. A subset of patients were referred to the Radboudumc after prior molecular testing carried out at other centres, either as part of routine practice or within clinical trials. Consequently, various platforms and NGS panels have been used for molecular profiling (Supplementary Table S1, available at https://doi.org/10.1016/j.esmoop.2026.106961).

In addition to these molecular techniques, immunohistochemistry (IHC) and *in situ* hybridisation (ISH) results were assessed to identify actionable biomarkers. These included anaplastic lymphoma kinase (ALK), AR, c-MET, epidermal growth factor receptor (EGFR), oestrogen receptor, HER2, and programmed death-ligand 1 (PD-L1). To determine the overall AR and HER2 status, the results from multiple specimens (primary tumours, locoregional recurrences, and distant metastasis) were pooled. The AR status was determined based on ≥1% nuclear staining, in accordance with the American Society of Clinical Oncology/College of American Pathologists (ASCO/CAP) guidelines on oestrogen and progesterone receptors.^29^ The HER2 status was defined as positive if IHC/ISH was positive according to the ASCO/CAP guidelines^21^ and/or if amplification or mutation(s) in the *ERBB2* gene were detected by NGS. Actionable molecular alterations were scored according to the European Society for Medical Oncology Scale of Clinical Actionability for molecular Targets (ESCAT).^30^

Molecular alterations detected by DNA NGS or gene fusion analysis were discussed at the molecular tumour board to consider treatment options. Alterations detected by IHC or ISH were not routinely discussed. Final treatment decisions were made by the treating physician, taking into account factors such as performance status, eligibility for clinical trials, treatment availability, and patient preferences.

### Definitions

Actionable and nonactionable pathogenic genetic aberrations detected by NGS and actionable targets identified by IHC and ISH were collectively defined as molecular alterations. MMT was defined as systemic therapy targeting molecular alterations present in the patient’s tumour. The best overall response was assessed according to RECIST v1.1.^31^ The ORR was defined as the proportion of patients achieving a complete response (CR) or a PR. The clinical benefit rate (CBR) was defined as the proportion of patients who achieved a CR, PR, or SD for ≥6 months. PFS was defined as the time from MMT initiation to radiological-confirmed disease progression or death by any cause. Patients without progression were censored at the date of last follow-up. OS was defined as the time from MMT initiation to death from any cause, with patients alive being censored at the date of last follow-up.

### Statistical analysis

As this study focuses on palliative systemic therapy, only patients with locally advanced and/or R/M SGC were included in our analyses. Descriptive statistics were used to report patient characteristics. Percentages of molecular alterations per subtype considered only patients whose DNA NGS panels covered the respective gene. PFS and OS were estimated using Kaplan–Meier statistics. PFS of SDC patients with and without specific molecular alterations was compared using the log-rank test. Statistical analyses were carried out using IBM SPSS Statistics 25 (v3.6.2), with *P* values of < 0.05 considered significant.

## Results

### Patients

**Figure 1:**
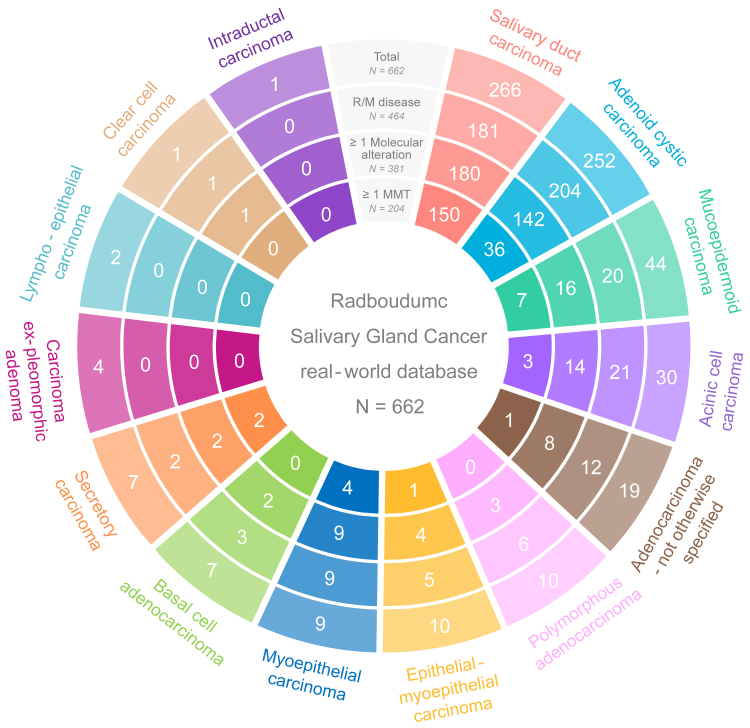
**Sunburst plot displaying an overview of patients in the Radboudumc Salivary Gland Cancer real-world database (*n* = 662).** Patients were classified into 14 histopathological subtypes. From the outer to inner ring, the plot shows the total number of patients, the number of patients diagnosed with R/M disease, the number of patients with R/M and one or more molecular alteration, and the number of patients treated with one or more MMT. MMT, molecular-matched therapy; R/M, recurrent and/or metastatic.

As of 3 July 2025, the Radboudumc Salivary Gland Cancer real-world database contained 662 patients. Figure 1 presents an overview of the cohort by histopathological subtype, including the prevalence of R/M disease (*n* = 464). The patient characteristics of the R/M cohort are summarised in Supplementary Table S2 (available at https://doi.org/10.1016/j.esmoop.2026.106961).

### Molecular alterations

At least one molecular alteration was detected in 381 of 464 patients with R/M disease (82%). Molecular alterations were observed in nearly all SDC cases (99%); when not taking into account AR positivity, which is almost universal in SDC, this rate was 85%. The rates of molecular alterations for AdCC, mucoepidermoid carcinoma, and acinic cell carcinoma were 70%, 80%, and 67%, respectively.

**Figure 2:**
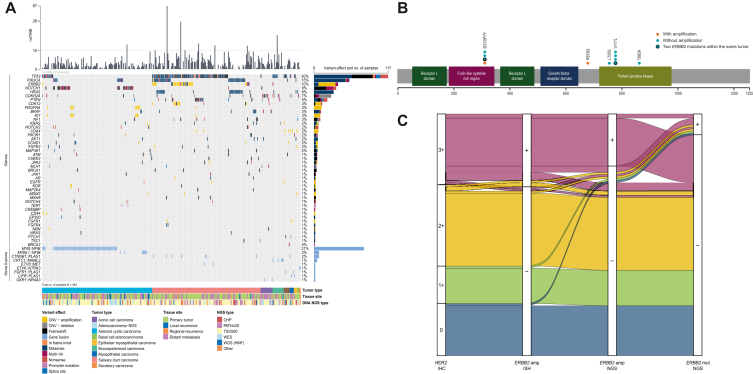
**Overview of molecular alterations in the cohort of patients with recurrent and/or metastatic (R/M) salivary gland cancer.** (A) Oncoplot showing genetic aberrations. Genes and gene fusions with a minimum of two occurrences in the total dataset are depicted. In total, samples from 384 patients were analysed. Data from 303 patients are shown as no genetic aberrations were detected in tumours of 71 patients, and 10 patients harboured genetic aberrations in genes not included in the figure. (B) Schematic representation of *ERBB2* mutations in patients with R/M salivary duct carcinoma. The coloured boxes illustrate the domains of the HER2 protein. Dots indicate mutation sites. The x-axis represents the amino acid position along the HER2 protein sequence. *ERBB2* amplification represents amplification detected by DNA NGS. (C) Alluvial plot showing the concordance between HER2 immunohistochemistry, *ERBB2* in situ hybridisation, *ERBB2* amplification determined by DNA NGS, and *ERBB2* mutations determined by DNA NGS in patients with recurrent and/or metastatic salivary duct carcinoma. Patients with available HER2 immunohistochemistry, *ERBB2* in situ hybridisation, and DNA next-generation sequencing were included in the analysis (*n* = 116). Amp, amplification; CHP, Cancer Hotspot Panel; CNV, copy number variant; HER2, human epidermal growth factor receptor 2; HMF, Hartwig Medical Foundation; IHC, immunohistochemistry; ISH, in situ hybridisation; mut, mutation; NGS, next-generation sequencing; nsTMB, non-synonymous tumour mutational burden; PATHv2D, Predictive Analysis for Therapy DNA gene panel version 2; TSO500, TruSight Oncology 500 actionable targets panel; WES, whole exome sequencing; WGS, whole-genome sequencing.

DNA NGS was available for 378 of 464 patients with R/M (81%). The primary reason for the absence of DNA NGS was the unavailability of the technique at the time of diagnosis (in 43/86 missing cases) (Supplementary Table S3, available at https://doi.org/10.1016/j.esmoop.2026.106961). Gene fusion detection assay data were available for 272 of 464 patients (58%). Figure 2A presents an overview of identified genetic aberrations in the cohort.

In SDC, *TP53* mutations were most prevalent (67%). Other frequent alterations in SDC included *PIK3CA* (30%) and *HRAS* (20%), with *PIK3CA*/*HRAS* mutations co-occurring in 19%. *ERBB2* alterations were identified in 29% of SDC cases, most often amplification (22%). Mutations of *ERBB2* were detected in 10 patients with SDC (7%), two of whom also exhibited *ERBB2* amplification by NGS. *ERBB2* mutations were mainly located in the protein tyrosine kinase domain (*n* = 6) and the furin-like cysteine rich region (*n* = 4) (Figure 2B). Common alterations in AdCC included *NOTCH1* mutations (24%) and gene fusions involving *MYB::NFIB* (57%) and *MYBL1::NFIB* (6%). In acinic cell carcinoma, *CDKN2A* alterations were frequent (47%), predominantly not only deletions (40%) but also mutations (20%). In mucoepidermoid carcinoma, *TP53* mutations (35%) and *CRTC1::MAML2* gene fusions (28%) were most common.

AR IHC data were available in 180 of 181 patients with R/M SDC (99%). Among these, 175 patients (97%) had a positive AR status. HER2 status was available for 165 of 181 patients with R/M SDC (91%), of whom 55 (33%) were HER2-positive and 110 (67%) were HER2-negative. When subdividing HER2-negative into HER2-low (IHC score 1+ or 2+) and HER2 zero (IHC score 0), 67 of 165 (41%) were HER2-low and 43 of 165 (26%) were HER2 zero. In 116 patients, HER2 status was assessed by IHC, ISH, and NGS, enabling evaluation of concordance among these techniques (Figure 2C). Tumours with IHC 3+ score were predominantly ISH-positive (32/34). Among these 32 IHC 3+/ISH+ tumours, *ERBB2* amplification by NGS was detected in 25 tumours. Both IHC 3+/ISH− tumours were also negative for *ERBB2* amplification by NGS, one harbouring an *ERBB2* mutation. Tumours with IHC 2+ score were largely ISH-negative (36/39), and all were negative for NGS amplification. All tumours with IHC 1+ (*n* = 18) and IHC 0 (*n* = 25) were negative by both ISH and NGS. *ERBB2* mutations were present not only in HER2-positive tumours, according to ASCO/CAP guidelines (*n* = 7), but also in some HER2-negative tumours (*n* = 3).

### Molecular-matched therapies

Among 381 patients with ≥1 molecular alteration, 204 (54%) were treated with at least one MMT. Among 180 patients with SDC and ≥1 molecular alteration, 150 (83%) received MMT, while 36 of 142 (25%) patients with AdCC and ≥1 molecular alteration received MMT. In this study, we first focus on AR–targeted and HER2–targeted therapies in SDC, followed by an overview of patients who received other MMTs.

#### AR-targeted therapies in SDC

A total of 126 patients with R/M SDC were treated with palliative AR-targeted therapy. Treatment was initiated from May 2001 to June 2025. The first course of AR-targeted therapy was administered as first-line in 110 patients (patient characteristics provided in Supplementary Table S4, available at https://doi.org/10.1016/j.esmoop.2026.106961), as second-line therapy in 12 patients, and as third-line therapy or beyond in four patients. The most common regimens were goserelin/bicalutamide (*n* = 81) and bicalutamide monotherapy (*n* = 37). Other regimens included goserelin/bicalutamide/dutasteride (*n* = 2), goserelin monotherapy (*n* = 1), leuprorelin/bicalutamide (*n* = 1), triptorelin/bicalutamide (*n* = 1), bicalutamide/palbociclib (*n* = 1), enzalutamide monotherapy (*n* = 1), and goserelin/enzalutamide (*n* = 1).

With any AR-targeted therapy as first-line systemic therapy, the mPFS was 5.3 months [95% confidence interval (CI) 3.5-7.2 months], and the mOS was 17.5 months (95% CI 14.7-20.4 months) (Supplementary Figure S1A and B, available at https://doi.org/10.1016/j.esmoop.2026.106961). Among patients receiving goserelin/bicalutamide as first-line therapy (*n* = 70), the mPFS was 6.1 months (95% CI 2.1-10.1 months), and the mOS was 19.6 months (95% CI 16.2-23.0 months) (Supplementary Figure S1C and D, available at https://doi.org/10.1016/j.esmoop.2026.106961). For first-line bicalutamide monotherapy (*n* = 35), mPFS was 3.5 months (95% CI 2.2-4.7 months), and mOS was 14.3 months (95% CI 10.1-18.4 months) (Supplementary Figure S1E and F, available at https://doi.org/10.1016/j.esmoop.2026.106961).

**Figure 3:**
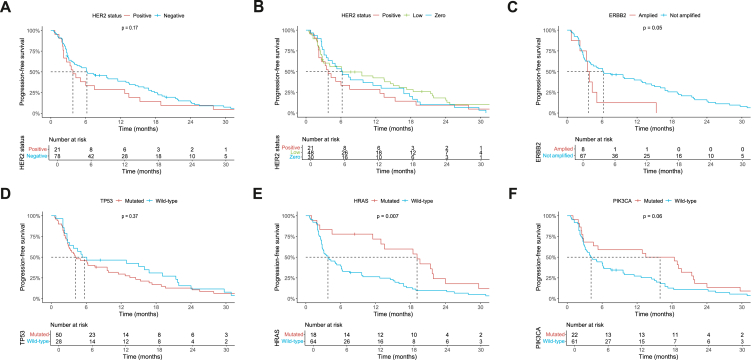
**Progression-free survival with first-line AR–targeted therapy in patients with recurrent and/or metastatic salivary duct carcinoma, stratified by molecular alterations.** (A) human epidermal growth factor receptor 2 (HER2) overall status positive versus negative. (B) HER2 overall status positive versus low versus zero. (C) *ERBB2* amplified versus not amplified, as determined by DNA next-generation sequencing. (D) *TP53*-mutant versus wild type. (E) *HRAS*-mutant versus wild type. (F) *PIK3CA*-mutant versus wild type. AR, androgen receptor.

With first-line AR-targeted therapy, HER2-positive patients (*n* = 21) had an mPFS of 3.8 months (95% CI 2.3-5.3 months), compared with 6.2 months (95% CI 2.3-10.1 months) in HER2-negative patients (*n* = 78; *P* = 0.17) (Figure 3A). After subdividing HER2-negative into HER2-low and HER2 zero, mPFS was 6.2 months (95% CI 0.0-13.2 months) for HER2-low (*n* = 48) and 6.1 months (95% CI 3.0-9.1 months) for HER2 zero (*n* = 30) (Figure 3B). mPFS of patients with *ERBB2* amplification detected by NGS (*n* = 8) was 3.5 months (95% CI 2.7-4.3 months), versus 6.2 months (95% CI 1.6-10.7 months) in patients without *ERBB2* amplification by NGS (*n* = 67; *P* = 0.05) (Figure 3C). Patients with *TP53*-mutant tumours (*n* = 50) had an mPFS of 4.1 months (95% CI 1.2-7.0 months), compared with 5.3 months (95% CI 0.0-16.0 months) in *TP53*–wild-type tumours (*n* = 28; *P* = 0.37) (Figure 3D). Interestingly, patients harbouring *HRAS* mutations (*n* = 18) achieved significantly longer mPFS (19.1 months; 95% CI 11.0-27.2 months) compared with *HRAS*–wild-type cases (*n* = 64; 3.8 months; 95% CI 2.0-5.6; *P* = 0.007) (Figure 3E). *PIK3CA* mutations were also associated with longer mPFS (*n* = 22; 13.5 months; 95% CI 0.0-29.1) compared with *PIK3CA*–wild-type tumours (*n* = 61; 4.1 months; 95% CI 2.4-5.9); however, this was not significant (*P* = 0.06) (Figure 3F). Interestingly, HER2-positivity and *HRAS* mutations appear largely mutually exclusive, with only few exceptions (Supplementary Figure S2, available at https://doi.org/10.1016/j.esmoop.2026.106961).

#### HER2-targeted therapies in SDC

**Figure 4:**
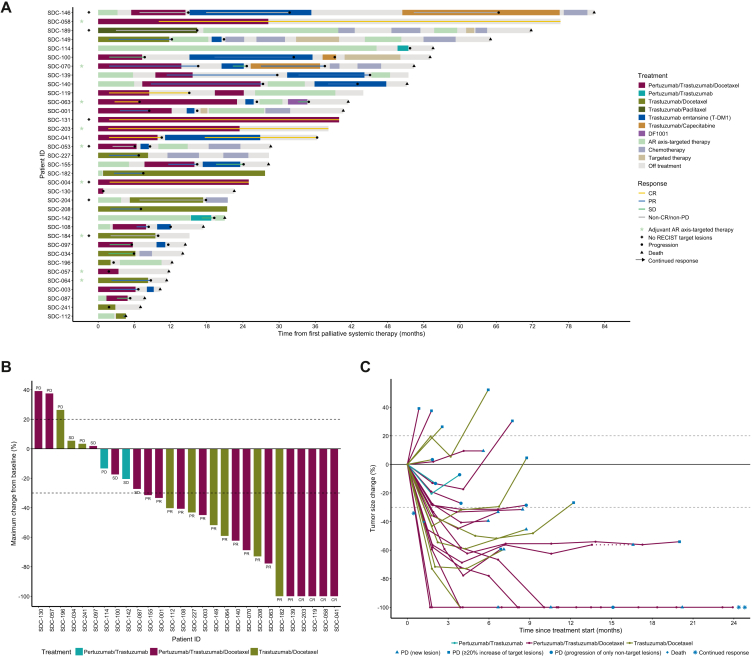
**Responses to HER2–targeted therapy in patients with HER2-positive salivary duct carcinoma (*n* = 35) according to RECIST version 1.1.** (A) Swimmer plot illustrating the sequence of systemic therapies and the response on HER2–targeted therapies. Bars represent individual patients and start at the initiation of the first palliative systemic therapy. (B) Waterfall plot demonstrating the maximum change in the sum of target lesions from baseline among patients with target lesions per RECIST version 1.1. Each bar represents one patient. Horizontal dashed lines indicate the threshold for PD (20% increase in the sum of target lesions) and partial response (30% decrease in the sum of target lesions). (C) Spider plot showing the change from baseline in sum of target lesions over time among patients with target lesions per RECIST version 1.1. Each line represents one patient. Dotted lines indicate time with ongoing response evaluation after treatment discontinuation. The upper horizontal dashed line indicates the threshold for progressive disease and the lower horizontal dashed line indicates the threshold for partial response. The two asterisks indicate two patients with continued response until 77 and 40 months. AR, androgen receptor; CR, complete response; HER2, human epidermal growth factor receptor 2; PD, progressive disease; PR, partial response; SD, stable disease.

A total of 35 patients with R/M HER2-positive SDC were treated with palliative HER2-targeted therapy. Patient characteristics are provided in Supplementary Table S4 (available at https://doi.org/10.1016/j.esmoop.2026.106961). Treatment was initiated from June 2011 to June 2024. Twenty-three patients received their first HER2–targeted therapy as first-line systemic treatment, 10 patients as second-line therapy, and 2 patients as third-line therapy (Figure 4A). The first HER2–targeted regimens per patient included pertuzumab/trastuzumab/docetaxel (*n* = 21), trastuzumab/docetaxel (*n* = 11), pertuzumab/trastuzumab (*n* = 2), and trastuzumab/paclitaxel (*n* = 1).

Across all first HER2-targeted regimens per patient, irrespective of treatment line, mPFS was 8.5 months (95% CI 6.4-10.6 months) and mOS was 40.7 months (95% CI 14.1-67.1 months) (Supplementary Figure S3A and B, available at https://doi.org/10.1016/j.esmoop.2026.106961). Among patients with evaluable target lesions (*n* = 28), the ORR was 61%, with a CR rate of 14% (Figure 4B and C). The CBR was 61% as well, as cases with SD for ≥6 months were not observed. In patients who received pertuzumab/trastuzumab/docetaxel as their first HER2–targeted regimen (*n* = 21), the mPFS was 8.7 months (95% CI 6.2-11.3 months) and the mOS was 41.5 months (95% CI 22.0-60.9 months) (Supplementary Figure S3C and D, available at https://doi.org/10.1016/j.esmoop.2026.106961). Both ORR and CBR were 71% in these patients, with a CR rate of 24%. In addition, two of three patients without target lesions who were treated with pertuzumab/trastuzumab/docetaxel achieved durable CR. With trastuzumab/docetaxel or trastuzumab/paclitaxel as first HER2–targeted regimen (*n* = 12), mPFS was 7.1 months (95% CI 3.6-10.6 months) and mOS was 65.1 months (95% CI 0.0-140.8 months) (Supplementary Figure S3E and F, available at https://doi.org/10.1016/j.esmoop.2026.106961). The ORR and CBR were both 67%, with no CR. In the two patients treated with pertuzumab/trastuzumab (SDC-142 and SDC-114), one had SD with progression after 3.8 months, and the other experienced progressive disease (PD) after 2.1 months (Figure 4A).

Five patients had known brain metastasis at the initiation of the first HER2-targeted therapy. Three received pertuzumab/trastuzumab/docetaxel: one (SDC-041) achieved CR followed by intracranial progression after 10.5 months and was alive at censoring at 54.0 months, one (SDC-003) achieved PR followed by systemic and intracranial progression after 6.7 months and deceased after 10.3 months, and one (SDC-146) showed non-CR/non-PD until intracranial progression after 9.5 months and deceased after 76.8 months. Furthermore, a patient (SDC-241) treated with trastuzumab/docetaxel developed intracranial progression at first evaluation after 1.8 months and deceased after 7.1 months, and a patient (SDC-142) treated with pertuzumab/trastuzumab had SD until cerebral progression after 3.8 months and deceased after 5.6 months.

Fourteen patients were treated with trastuzumab emtansine as second-line systemic treatment or beyond: 13 after pertuzumab/trastuzumab/docetaxel and 1 after trastuzumab/docetaxel (Figure 4A). Among patients with evaluable target lesions (*n* = 12), the ORR and CBR were both 33%, with a CR rate of 8%. Among the two patients without target lesions, one had non-CR/non-PD until 16.6 months (SDC-146), while the other had PD after 1.7 months (SDC-053). After pertuzumab/trastuzumab/docetaxel and trastuzumab emtansine, three patients were treated with trastuzumab/capecitabine. Among those, one achieved PR until 12.5 months (SDC-070), one showed non-CR/non-PD until 16.0 months (SDC-146), and one experienced PD after 2.0 months (SDC-100).

Finally, one patient with negative HER2 status per ASCO/CAP guidelines (HER2 IHC score 2+ and *ERBB2* ISH-negative) received the combination of vic-trastuzumab duocarmazine and niraparib as third-line systemic treatment in a phase I trial (NCT04235101). The first response evaluation after 1.6 months demonstrated PD.

#### Other MMTs

Table 1 provides an overview of patients (*n* = 73) who received MMTs other than AR-targeted and HER2-targeted therapy in SDC, including ESCAT scores, PFS, and best overall response. In this section, we highlight several cases of particular interest.

**Table 1:** Overview of molecular-matched therapies in salivary gland cancer other than AR–targeted therapy and HER2–targeted therapy in SDC, including ESCAT scores,^30^ progression-free survival, and best overall response.

Diagnosis	Target	ESCAT score^30^	Treatment	Systemic treatment line	Progression-free survival (months)	Best overall response	Treatment access type	Patient ID
AcCC	AR expression	III-A	Bicalutamide	1	2.2	PD	Reimbursed care	AcCC-004
	c-MET expression	III-A	Cabozantinib	1	6.6^a^	SD	Clinical trial^25^	AcCC-008
	HER2 expression	III-A	Pertuzumab/trastuzumab/docetaxel	2	4.0	PR	Compassionate use	AcCC-004
	MSI high	I-C	Nivolumab	1	5.8	SD	Reimbursed care	AcCC-018
AdCC	*ATM* mut	III-A	Olaparib	2	11.6	SD	Clinical trial^32^	AdCC-038
	*BRAF* G464A mut	III-A	Regorafenib	3	16.6	SD	Clinical trial^32^	AdCC-128
	c-MET expression	III-A	Cabozantinib	1	12.8^a^	SD	Clinical trial^25^	AdCC-010
					12.8^a^	PR	Clinical trial^25^	AdCC-138
					12.6^a^	SD	Clinical trial^25^	AdCC-063
					12.4^a^	SD	Clinical trial^25^	AdCC-018
					9.9	SD	Clinical trial^25^	AdCC-088
					7.3	SD	Clinical trial^25^	AdCC-019
					4.8^a^	SD	Clinical trial^25^	AdCC-044
					3.4	SD	Clinical trial^25^	AdCC-053
					2.1^a^	SD	Clinical trial^25^	AdCC-003
					2.0^a^	SD	Clinical trial^25^	AdCC-032
					0.9^a^	NE	Clinical trial^25^	AdCC-118
					0.8^a^	NE	Clinical trial^25^	AdCC-196
				2	18.3	SD	Compassionate use	AdCC-028
					10.9^a^	SD	Clinical trial^25^	AdCC-128
					7.9b	SD	Clinical trial^25^	AdCC-035
					5.7^a^	SD	Clinical trial^25^	AdCC-087
				3	5.0^a^	SD	Clinical trial^25^	AdCC-038
	*EGFR* mut	III-A	Amivantamab	2	2.7^b^	SD	Compassionate use	AdCC-106
			Osimertinib	1	16.9	SD	Clinical trial^28^	AdCC-106
	Oestrogen receptor expression	III-A	Tamoxifen	1	1.0	PD	Reimbursed care	AdCC-169
	*NOTCH1* mut	IV-A	Osugacestat (AL101)	1	3.1	SD	Clinical trial (NCT03691207)	AdCC-095
					2.0	PD	Clinical trial (NCT03691207)	AdCC-036
					1.4	PD	Clinical trial (NCT03691207)	AdCC-111
	*NOTCH1* mut and *NOTCH2* mut	IV-A	Osugacestat (AL101)	1	9.5	SD	Compassionate use	AdCC-149
	*NOTCH2* mut	IV-A	Osugacestat (AL101)	1	3.7	SD	Clinical trial (NCT03691207)	AdCC-102
	*PDGFRA* amplification	III-A	Sunitinib	2	3.9	SD	Clinical trial^32^	AdCC-200
	*PDGFRA* and *KIT* co-amplification	III-A	Sunitinib	1	4.1	SD	Clinical trial^32^	AdCC-136
				3	5.7	SD	Clinical trial^32^	AdCC-028
	PSMA expression	V	PSMA-targeted T-cell enhancer CB307	1	14.7^a^	SD	Clinical trial^27^	AdCC-057
		III-A	Lutetium-177-PSMA-I&T	1	11.6	SD	Compassionate use	AdCC-072
					26.9	SD	Clinical trial^26^	AdCC-140
					20.8	SD	Clinical trial^26^	AdCC-114
					9.9	SD	Clinical trial^26^	AdCC-049
					7.6	SD	Clinical trial^26^	AdCC-081
					6.7	SD	Clinical trial^26^	AdCC-007
					2.4	PD	Clinical trial^26^	AdCC-069
					1.4	PD	Clinical trial^26^	AdCC-031
				2	2.6	PD	Clinical trial^26^	AdCC-196
				3	2.3	PD	Clinical trial^26^	AdCC-092
Adeno–NOS	*EGFR* amplification	III-A	Panitumumab	2	7.8	SD	Clinical trial^32^	Adeno-NOS-002
EMC	*PIK3RA* mut and monoallelic loss of *PIK3R1*	III-A	Alpelisib	1	10.2	SD	Clinical trial^32^	EMC-004
MECA	AR expression	III-A	Bicalutamide/Goserelin	3	1.5	PD	Reimbursed care	MECA-002
	*CDK4* amplification	V	Palbociclib	2	7.0	SD	Compassionate use	MECA-003
	*CDKN2A* loss	V	Ribociclib	1	1.4	PD	Clinical trial^32^	MECA-005
	c-MET expression	III-A	Cabozantinib	1	7.6	SD	Clinical trial^25^	MECA-001
	EGFR expression	III-A	Cetuximab/Carboplatin	2	1.8	PD	Reimbursed care	MECA-002
MEC	ALK expression	III-A	Crizotinib	6	2.5^b^	SD	Compassionate use	MEC-016
	c-MET expression	III-A	Cabozantinib	2	0.7^a^	NE	Clinical trial^25^	MEC-005
	*FGFR3* amplification	X	Lenvatinib	1	30.9	SD	Clinical trial^32^	MEC-011
	*CRTC1::MAML2* gene fusion leading to activated EGFR signalling	IV-A	Cetuximab	1	3.0	PD	Reimbursed care	MEC-029
		IV-A	Erlotinib	1	7.3	SD	Compassionate use	MEC-028
				2	5.1	SD	Compassionate use	MEC-002
	PD-L1 expression	III-A	Pembrolizumab	1	2.4	PD	Reimbursed care	MEC-005
	TMB high	I-C	Ipilimumab/Nivolumab	1	24.0^c^	PR	Clinical trial^32^	MEC-034
SC	*ETV6*::*NTRK3* gene fusion	I-C	Larotrectinib	1	39.8^c^	CR	Reimbursed care	SC-001
					4.5	PR	Reimbursed care	SC-003
SDC	*BRAF* D594N mut	IV-A	Trametinib	3	0.5^b^	NE	Clinical trial^32^	SDC-002
	*BRAF* V600E mut	III-A	Vemurafenib/Cobimetinib	1	11.9	CR	Clinical trial^32^	SDC-043
				3	25.3	PR	Clinical trial^32^	SDC-148
					11.1	PR	Clinical trial^32^	SDC-219
	*CDKN2A* loss	V	Ribociclib	2	3.0	SD	Clinical trial^32^	SDC-090
	c-MET expression	III-A	Cabozantinib	1	3.7	SD	Clinical trial^25^	SDC-021
				2	5.5^b^	SD	Clinical trial^25^	SDC-002
				3	1.1^a^	NE	Clinical trial^25^	SDC-001
				5	8.5	PR	Clinical trial^25^	SDC-100
				6	7.2^a^	SD	Clinical trial^25^	SDC-149
	*ERBB2* mut	IV-A	DF1001	4	3.7	PD	Clinical trial (NCT04143711)	SDC-063
	*FGFR2* mut	IV-A	Lenvatinib	1	5.8	PR	Clinical trial^32^	SDC-210
	*HRAS* mut	III-A	Tipifarnib	4	5.5	SD	Compassionate use	SDC-048
	*MAP2K4* mut	X	Alpelisib	1	3.7	SD	Clinical trial^32^	SDC-069
	PD-L1 expression	III-A	Pembrolizumab	1	1.7	PD	Compassionate use	SDC-039
	*PIK3CA* mut	III-A	Alpelisib	2	1.2^b^	PD	Clinical trial^32^	SDC-127
				5	1.1	PD	Compassionate use	SDC-048
		III-A	Temsirolimus followed by Everolimus	3	1.8	SD	Compassionate use	SDC-154
	PSMA expression	III-A	Lutetium-177-PSMA-I&T	3	2.5	PD	Clinical trial^26^	SDC-044
				4	0.4	PD	Clinical trial^26^	SDC-109
	TMB high	I-C	Ipilimumab/Nivolumab	1	3.0	PD	Clinical trial^32^	SDC-074

AcCC, acinic cell carcinoma; AdCC, adenoid cystic carcinoma; Adeno–NOS, adenocarcinoma–not otherwise specified; AR, androgen receptor; c-MET, c-mesenchymal-epithelial transition factor; CR, complete response; EGFR, epidermal growth factor receptor; EMC, epithelial–myoepithelial carcinoma; ESCAT, ESMO Scale for Clinical Actionability of molecular Targets; HER2, human epidermal growth factor receptor 2; MECA, myoepithelial carcinoma; MEC, mucoepidermoid carcinoma; mut, mutation; MSI, microsatellite instability; NE, not evaluable; PD, progressive disease; PD-L1, programmed death-ligand 1; PR, partial disease; PSMA, prostate-specific membrane antigen; SC, secretory carcinoma; SD, stable disease; TMB, tumour mutational burden.

a Progression-free survival censored at closure of the trial.^25^

b Progression-free survival censored at treatment discontinuation due to toxicity.

c Progression-free survival censored at last response evaluation due to continued response at data cutoff.

Three patients with SDC were treated with vemurafenib/cobimetinib, targeting *BRAF* V600E mutations, in a basket trial.^32^ One patient achieved a durable CR with first-line vemurafenib/cobimetinib. After 11.9 months, brain metastases were detected. It was unclear whether these were already present pretreatment. The patient underwent whole-brain radiotherapy twice, while continuing vemurafenib/cobimetinib, and maintained extracranial CR. After 21.8 months, the brain metastases progressed, leading to discontinuation of systemic therapy. The other two patients treated with vemurafenib/cobimetinib demonstrated PR, with one maintaining durable PR until 25.3 months and the other until 11.1 months.

Primary tumours of two patients with secretory carcinoma harboured *ETV6::NTRK3* gene fusions, and both patients subsequently received first-line treatment with larotrectinib. Durable CR—ongoing at data cutoff at 39.8 months—was observed in one patient. In the other patient, PR was observed until 4.5 months.

Another remarkable response was achieved in a patient with mucoepidermoid carcinoma with high tumour mutational burden (TMB; 21.9 mut/Mb). Treatment with ipilimumab/nivolumab in a basket trial^32^ resulted in ongoing PR at data cutoff at 24.0 months.

## Discussion

This study provides an overview of the real-world application and the effectiveness of MMTs in SGC. The majority of patients with R/M SGC—82% in our cohort—exhibit one or more molecular alteration, offering therapeutic options for this disease setting where systemic treatment options are limited. In our cohort, 54% of patients with a molecular alteration received at least one MMT. As the number of druggable molecular targets continues to expand, an increasing proportion of patients with SGC will become eligible for personalised systemic treatments. Our findings, demonstrating clinical benefit in a subset of patients who would otherwise have had few or no systemic treatment options, suggest that comprehensive molecular profiling and, if available, subsequent MMT may be considered in patients with SGC. Moreover, our study suggests that *HRAS* mutations may serve as a predictive biomarker for AR-targeted therapy in SDC, as patients with *HRAS*-mutant SDC showed superior PFS than patients with *HRAS*–wild-type SDC.

AR-targeted therapy has demonstrated efficacy exclusively in AR-positive SDC and adenocarcinoma not otherwise specified and represents standard palliative treatment for R/M AR-positive SDC.^7^^,^^8^ In our real-world cohort, mPFS with any first-line AR-targeted therapy was 5.3 months and mOS was 17.5 months. With first-line goserelin/bicalutamide, mPFS was 6.1 months and mOS was 19.6 months, lower than in a phase II trial with leuprorelin/bicalutamide, where mPFS of 8.8 months and mOS of 30.5 months were observed.^16^ By contrast, another phase II study with triptorelin/bicalutamide reported lower mPFS of 4.0 months.^33^ More in line with our findings, a retrospective study by Kawakita et al.^34^ reported mPFS of 6 months and mOS of 27 months with leuprorelin/bicalutamide in HER2-positive SDC. Overall, our findings confirm that AR-targeted therapy provides clinically meaningful benefit for R/M AR-positive SDC in a real-world setting.

Nevertheless, routinely available biomarkers to predict the clinical benefit from AR-targeted therapy are currently lacking. Only RNA-based expression levels of *SRD5A1* and AR pathway activity scores have been demonstrated to predict the clinical benefit from AR-targeted therapy in SDC.^35^^,^^36^ In our cohort (*n* = 82, *HRAS*-mutant *n* = 18), first-line AR-targeted therapy was associated with a significantly longer PFS in patients with *HRAS*-mutant SDC than that with *HRAS*–wild-type cases (mPFS 19.1 versus 3.8 months). A possible explanation may involve activating *HRAS* mutations that drive constitutive MAPK signalling, leading to AR activation, as shown in prostate cancer models.^37^ Such crosstalk may result in more reliance of *HRAS*-mutant SDC on AR signalling, thereby increasing their susceptibility to AR-targeted therapies. Alternatively, the superior outcomes of *HRAS*-mutant cases could partially reflect their mutual exclusivity with *ERBB2* alterations,^38^ although HER2 status in our cohort was not significantly associated with PFS. To our knowledge, the superior clinical benefit of AR-targeted therapy in patients with *HRAS-*mutant SDC has not been reported previously. Validation in external cohorts is warranted and currently ongoing before *HRAS* mutation status can be applied as a predictive biomarker for AR-targeted therapy in clinical practice.

In HER2-positive SDC, HER2-targeted therapy can be recommended before AR-targeted therapy.^34^ Our real-world data demonstrated mPFS of 8.5 months, mOS of 40.7 months, and ORR of 61% with HER2-targeted therapy. These outcomes are comparable with those of a phase II trial and a retrospective study evaluating trastuzumab/docetaxel.^17^^,^^34^ Our results suggest that combining two HER2-targeted regimens (pertuzumab and trastuzumab) combined with docetaxel may yield more durable and deeper responses than the combination of trastuzumab and docetaxel. Two patients treated with dual HER2-targeted therapy (pertuzumab/trastuzumab) without chemotherapy demonstrated poor outcomes, indicating that chemotherapy may be advisable when patients are clinically fit. Overall, our real-world findings confirm the effectiveness of HER2-targeted therapies and support the investigation of next-generation HER2-targeted agents in SDC.

The armamentarium of HER2-targeted therapies has expanded, with trastuzumab–deruxtecan demonstrating efficacy in HER2-low expressing (IHC score 1+ or 2+) and *ERBB2*-mutant cancers,^39^^,^^40^ while the HER2 tyrosine kinase inhibitor zongertinib showed efficacy in *ERBB2*-mutant non-small-cell lung cancer.^41^^,^^42^ In our SDC cohort, the prevalence of HER2-low was 41%, similar to reported rates of 35%-57% in other SDC cohorts.^43^^,^^44^ *ERBB2* mutations were detected in 7% of our SDC cohort, slightly lower than reported rates of 10%-11% in other SDC cohorts.^45^^,^^46^ *ERBB2* mutations were present in both HER2-positive (*n* = 7) and HER2-negative (*n* = 3) tumours. Most mutations were located within the protein tyrosine kinase domain, the region associated with the highest sensitivity to zongertinib.^41^ These findings prompt investigation of trastuzumab–deruxtecan and zongertinib in HER2-low expressing and *ERBB2*-mutant SDC.

The *ETV6::NTRK3* gene fusion is a distinctive molecular alteration in secretory carcinoma.^2^ In our real-world cohort, seven patients with secretory carcinoma have been included, of which two developed R/M disease. Both tumours harboured an *ETV6::NTRK3* gene fusion, which was targeted with larotrectinib, a selective TRK-inhibitor. This resulted in objective responses in both patients, including continued CR at 39.8 months. Larotrectinib has been shown to be highly effective in TRK fusion-positive SGC, with ORR of 92%.^14^ These results highlight the importance of *NTRK* gene fusion analysis in all R/M SGC cases. These results highlight the importance of *NTRK* gene fusion analysis in all R/M SGC cases to prevent misdiagnosis and to identify all patients who may benefit from TRK inhibitors.

*BRAF-*targeted treatment has previously demonstrated encouraging clinical activity across various *BRAF* V600E-mutant nonmelanoma cancers, including a PR in one patient with SDC.^47^ Additionally, two case reports have described responses to dabrafenib/trametinib in SDC.^48^ In our cohort, we report for the first time three patients with *BRAF* V600E-mutant SDC, treated with the *BRAF*/*MEK*-inhibitors vemurafenib/cobimetinib. All three patients achieved durable responses, one CR until 11.9 months and two PRs until 25.3 and 11.1 months. Despite its rarity, our findings underscore the importance of assessing the *BRAF* mutation status in patients with R/M SDC and support *BRAF*-targeted treatment in *BRAF* V600E-mutant cases.

Immune checkpoint blockade has shown promise for non-AdCC SGC, with infrequent but exceptional responses.^49^ In our cohort, two patients received ipilimumab/nivolumab for TMB-high tumours. One patient with mucoepidermoid carcinoma (21.9 mut/Mb) achieved a durable PR, ongoing at 24.0 months. While the overall efficacy of immune checkpoint blockade in unselected patients with SGC is limited, the exceptional responses in select cases highlight the need for predictive biomarkers, such as high TMB, microsatellite instability, and high T-cell infiltration.^50^ However, the limited evidence available for immune checkpoint blockade in SGC hinder the definition of SGC-specific biomarker cutoffs. As universal TMB thresholds across various malignancies appear to be insufficient,^51^ lower thresholds may be considered for subtypes with more favourable immunogenic profiles, such as SDC,^52^ until SGC-specific biomarker cutoffs have been established.

In our cohort, AdCC represents the subtype with the largest number of patients with R/M disease (*n* = 204). Although molecular alterations were detected in 142 of these patients, most were currently considered nonactionable, including *MYB-NFIB* gene fusions and *TP53* mutations. As a result, only 36 patients with AdCC received MMT. Among these, 17 patients were treated with cabozantinib and 10 with prostate-specific membrane antigen–targeted radioligand therapy, both with low clinical benefit, as previously described.^25^^,^^26^ Five patients received the pan-NOTCH inhibitor osugacestat, which also showed limited activity, consistent with a larger cohort of patients with AdCC treated with NOTCH inhibitors.^18^ Best overall responses with other MMTs were limited to SD. While disease stabilisation might indicate antitumour activity, it could also reflect the indolent clinical behaviour of a subgroup of AdCC tumours.^53^ Therefore, despite the considerable number of patients with AdCC that received MMT in this cohort, none demonstrated meaningful antitumour activity. This leaves a large unmet need for effective systemic therapies in AdCC, underscoring the importance of clinical trials of novel agents with a strong biological rationale, such as agents targeting *MYB* gene fusions, NOTCH mutations, and B7-H4.^54^

This is an unprecedented study providing one of the most comprehensive real-world data on clinical outcomes of MMTs in SGC. The real-world setting provides valuable insights into routine practice, complementing the evidence obtained from clinical trials. However, the real-world design also introduces inherent limitations, as data were collected retrospectively, with molecular testing, treatment approaches, and response evaluation varying over time and across centres. Specifically, various DNA NGS platforms and panels were used, some lacking coverage of certain molecular alterations and gene fusion analysis was available only in a subset of patients. This may have resulted in undetectability of certain alterations, and alteration frequencies should therefore not be compared between subtypes. Moreover, some patients with actionable molecular alterations did not receive MMT, due to factors including poor performance status, ineligibility for clinical trials, treatment availability, physician choice, and patient preferences. Furthermore, the 1% cutoff for AR positivity in SDC has been an institutional choice for this study, given the absence of international consensus about this threshold. Additionally, toxicity data were not collected because these were not systematically registered. Finally, although the cohort represents a relatively large SGC population considering the rarity of the disease, the number of cases per subtype remains limited, particularly for the ultrarare subtypes.

### Conclusions

In this real-world cohort, 82% of patients with R/M SGC exhibited at least one molecular alteration, and 54% of them received at least one MMT. Our data indicate that comprehensive molecular testing in patients with SGC may render them eligible to MMT if available, particularly in subtypes lacking standard systemic therapies. A subset of patients demonstrated clinical benefit from treatment with MMTs, with some experiencing durable responses.
