# Supplementary material for: Real-world effectiveness of molecular-matched therapies in salivary gland cancer
Source: ESMO Open. 2026 Apr 30;11(5):106961. doi: 10.1016/j.esmoop.2026.106961 (PMC13145383; doi:10.1016/j.esmoop.2026.106961)
Supplement: Supplementary Figures S1-S3 and Tables S1-S4 [file mmc1.pdf]

Supplementary Tables & Figures

ESMO Open

Real-world effectiveness of molecular-matched therapies in salivary gland cancer

Author names

Jetty A.M. Weijers[a]*, Niels J. van Ruitenbeek[a]*, Adriana C.H. van Engen-van Grunsven[b], Chantal M.L. Driessen[a], Lot A. Devriese[c], Marije Slingerland[d], Ann Hoeben[e], Sjoukje F. Oosting[f], Willem H. Schreuder[g], Aniel Sewnaik[h], Sjoerd van Helvert[b], Jack A. Schalken[i], Gerald W. Verhaegh[i], Carla M.L. van Herpen[a]

* Authors contributed equally

Affiliations

[a] Department of Medical Oncology, Radboud Institute for Medical Innovation, Radboud university medical center, Nijmegen, The Netherlands

[b] Department of Pathology, Radboud Institute for Medical Innovation, Radboud university medical center, Nijmegen, The Netherlands

[c] Department of Medical Oncology, University Medical Center Utrecht, Utrecht, The Netherlands

[d] Department of Medical Oncology, Leiden University Medical Center, Leiden, The Netherlands

[e] Division of Medical Oncology, Department of Internal Medicine, Maastricht UMC+ Comprehensive Cancer Centre, GROW-School of Oncology and Reproduction, Maastricht University Medical Centre+, Maastricht, The Netherlands

[f] Department of Medical Oncology, University Medical Centre Groningen, University of Groningen, Groningen, the Netherlands

[g] Department of Head and Neck Surgery & Oncology, The Netherlands Cancer Institute, Amsterdam, The Netherlands

[h] Department of Otorhinolaryngology, Head and Neck Surgery, Erasmus Medical Centre, Rotterdam, The Netherlands

[i] Department of Urology, Radboud Institute for Medical Innovation, Radboud university medical center, Nijmegen, The Netherlands

Corresponding author

Prof. dr. Carla M.L. van Herpen

Radboud university medical center

Department of Medical Oncology

P.O. box 9101, Nijmegen, The Netherlands

Carla.vanHerpen@radboudumc.nl

Phone number: + 31 24 3610353

Supplementary Table S1. Overview of analysed genes per next-generation sequencing panel.

**NGS panel**	***TP53* mut**	***HRAS* mut**	***PIK3CA* mut**	***ERBB2* mut**	***ERBB2* CNA**	***CDKN2A* mut**	***CDKN2A* CNA**	***NOTCH1* mut**	**Genes analysed**
General diagnostics panel version 5.1 (Erasmus Medical Centre)	No	Yes	Yes	Yes	No	No	No	No	Coding sequence: *CDKN2A* (coverage low design 90%), *PTEN* (94%), *TP53* (100%).<br>Mutation hotspots: *AKT1* (exon 3), *ALK* (20–25), *APC* (4), *ARID1A*, *ARID2* (11), *BRAF* (11, 15), *CTNNB1* (3, 7, 8), *EGFR* (18–21), *ERBB2* (19, 20), *ERBB4* (8, 10), *FGFR2* (4, 12), *FGFR3* (7, 9, 12), *FGFR4* (7), *FOXL2* (1), *GNA11* (4, 5), *GNAQ* (4, 5), *GNAS* (8), *HRAS* (2), *IDH1* (4), *IDH2* (4), *KIT* (8, 9, 11, 13, 14, 17), *KRAS* (2–4), *MAP2K1* (2, 3), *MET* (14), *MYD88* (5), *NRAS* (2–4), *NTRK1* (13), *PDGFRA* (12, 18), *PIK3CA* (10, 21), *POLD1 (12), POLE (9, 13), RAF1 (7), RET (8, 16), RER3 (3, 4, 9), ROS1 (31, 34, 41), SMAD4 (9, 12), STK11 (4, 5, 8).*<br><br>Non-coding sequence: TERT promoter<br>Detection limit: 20% neoplastic cells
Cancer Hotspot gene panel version 1.0 (Radboud university medical centre)	Yes	Yes	Yes	Yes	No	No	No	No	Cancer Hotspot gene panel version 1.0 (Radboud university medical centre)
Cancer Hotspot gene panel version 2.0 (Radboud university medical centre)	Yes	Yes	Yes	Yes	Yes	Yes	Yes	Yes	Cancer Hotspot gene panel version 2.0 (Radboud university medical centre)
Cancer Hotspot Panel version 2Plus (University Medical Centre Utrecht)	Yes	Yes	Yes	Yes	Yes	Yes	Yes	Yes	Cancer Hotspot Panel version 2Plus (University Medical Centre Utrecht)
Cancer Hotspot Panel version 4 (Leiden University Medical Centre)	Yes	Yes	Yes	Yes	Yes	Yes	Yes	Yes	*ARAF* (exons 2, 4-6 , 7, 9, 10, 11, 15, 16), *CD79B* (5, 6), *CIC* (5), *CTNNB1* (1, 2, 4, 7, 8, 12, 15), *EIF1AX* (1, 3-6), *ERBB3* (23), *KRAS* (2-4), *NRAS* (2-4), *HRAS* (2-3), *BRAF* (6, 11,15), *EGFR* (3, 7, 15, 18-21), *GNAQ* (4, 5), *GNAS* (8-9), *H3F3A* (2), *H3F3B* (2), *IDH1* (4), *IDH2* (4), *KIT* (2, 9-18), *MAP2K1* (2-4, 6, 7, 11), *MAP2K2* (2, 3), *MAP2K4* (2, 4, 5, 9), *MAP3K1* (5, 8, 14, 17, 20), *MDM2* (3, 4, 6, 7, 8), *MED12* (2), *MYD88* (3b, 5), *MUTYH* (7, 13), *PDGFRA* (12, 14, 15, 18, 23), *PDGFRB* (12, 14), *PIK3CA* (2, 5, 6-10, 14, 18, 21), *POLE* (9-14), *RET* (10-12, 15, 16), *TP53* (1-11).<br><br>*Mutation hotspots: ABL1, AKT1, ALK, APC, ATM, CARD11, CD79A, CDK4, CDH1, CDKN2A, CSF1R, CTNNB1, ERBB2, ERBB4, EZH2, FBXW7, FGFR1, FGFR2, FGFR3, FLT3, FOXL2, GNA11, HNF1A, JAK2, JAK3, KDR, MET, MLH1, MPL, MYC, NOTCH1, NPM1, PTEN, PTK2, PTPN11, RB1, SMAD4, SMARCB1, SMO, SRC, STK11, VHL.*
Cancer Hotspot panel version 6 (Leiden University Medical Centre)	Yes	Yes	Yes	Yes	Yes	Yes	Yes	Yes	Cancer Hotspot panel version 6 (Leiden University Medical Centre)
Centre for Personalized Cancer Treatment gene panel	Yes	Yes	Yes	Yes	Yes	Yes	Yes	Yes	Mutation hotspots: *ABL1, AKT1, ALK, APC, ATM, BRAF, BRCA1, BRCA2, CDH1, CDKN2A, CSF1R, CTNNB1, EGFR, ERBB2, ERBB4, EZH2, FBXW7, FGFR1, FGFR2, FGFR3, FLT3, GNA11, GNAQ, GNAS,*

									HNF1A, HRAS, IDH1, IDH2, JAK2, JAK3, KDR, KIT, KRAS, MET, MLH1, MPL, NOTCH1, NPM1, NRAS, PDGFRA, PIK3CA, PTEN, PTPN11, RB1, RET, SMAD4, SMARCB1, SMO, SRC, STK11, TP53, VHL.
EGFR and HER2 signal transduction gene panel version 2.0 and melanoma/GIST panel version 2.0 (Radboud university medical centre)	No	Yes	Yes	Yes	Yes	No	No	No	*AKT1* (codon 17), *BRAF* (600), *ERBB2* (772-781), *GNA11* (183, 209), *GNAQ* (183, 209), *HRAS* (12, 13, 59, 61), *KRAS* (12, 13, 59, 61, 117, 146), *NRAS* (12, 13, 59, 61, 117, 146), *PIK3CA* (538-548 and 1040-1050).<br><br>*EGFR* (exons 18-21), *KIT* (8, 9, 11, 13, 14, 17), *PDGFRA* (12, 14, 18).
Hartwig Medical Foundation gene panel	Yes	Yes	Yes	Yes	Yes	Yes	Yes	Yes	Hartwig Medical Foundation gene panel
Ion AmpliSeq Cancer Hotspot panel version 2	Yes	Yes	Yes	Yes	Yes	Yes	Yes	Yes	Mutation hotspots: *ABL1, AKT1, ALK, APC, ATM, BRAF, CDH1, CDKN2A, CSF1R, CTNNB1, EGFR, ERBB2, ERBB4, EZH2, FBXW7, FGFR1, FGFR2, FGFR3, FLT3, GNA11, GNAS, GNAQ, HNF1A, HRAS, IDH1, JAK2, JAK3, IDH2, KDR, KIT, KRAS, MET, MLH1, MPL, NOTHC1, NPH1, NRAS, PDGFRA, PIK3CA, PTEN, PTPN11, RB1, RET, SMAD4, SMARCB1, SMO, SRC, STK11, TP53, VHL.*
Ion AmpliSeq panel Lung version 1	Yes	No	Yes	Yes	Yes	Yes	Yes	Yes	*ABL1* (exons 4-7), *AKT1* (3, 6), *ALK* (15, 21-25), *BRAF* (11, 15), *CDKN2A* (2), *CTNNB1* (3), *EGFR* (12, 18-21), *ERBB2* (8, 19-21), *FGFR1* (4, 7), *FGFR2* (6, 8, 11), *FGFR3* (7, 9, 14, 16, 18), *IDH1* (4), *IDH2* (4), *JAK2* (14), *KIT* (2, 9-11, 13-15, 17, 18), *KRAS* (2-4), *MAP2K1* (1-3, 6), *MET* (2, 11, 14, 16, 19), *NOTCH1* (26, 27, 34), *NRAS* (2-4), *PIK3CA* (10, 14, 21), *RB1* (4, 6, 10, 11, 14, 17, 18, 20-22), *RET* (9-11, 13, 15, 16), *RIT1* (4, 5), *ROS1* (35-38, 41), *STK11, TP53.*
Ion Torrent Oncomine focus DNA gene panel	No	Yes	Yes	Yes	Yes	No	No	No	Mutation hotspots: *AKT1, ALK, AR, BRAF, CDK4, CTNNB1, DDR2, EGFR, ERBB2, ERBB3, ERBB4, ESR1, FGFR2, FGFR3, GNA11, GNAQ, HRAS, IDH1, IDH2, JAK1, JAK2, JAK3, KIT, KRAS, MAP2K1, MAP2K2, MET, MTOR, NRAS, PDGFRA, PIK3CA, RAF1, RET, ROS1, SMO.*
Maxima RNAseq gene panel version 24.1	Yes	Yes	Yes	Yes	No	No	No	Yes	Maxima RNAseq gene panel version 24.1
Oncomine Comprehensive Assay plus panel (Leiden University Medical Centre)	Yes	Yes	Yes	Yes	Yes	Yes	Yes	Yes	Oncomine Comprehensive Assay plus panel (Leiden University Medical Centre)
Oncopanel version 1 (Amsterdam University Medical Centre)	Yes	Yes	Yes	Yes	Yes	Yes	Yes	No	*ABL1* (exons 4-7), *AKT1* (3, 6), *AKT2, AKT3, ALK* (22, 23, 25), *APC, ARAF,* BRAF (11, 15), *CCND1* (1, 2, 4), *CDK4* (2, 4, 8, + amp), *CDK6* (2, 5, 8, + amp), *CDKN2A, CRKL, CSF1R* (7, 22), *DLL3, EGFR* (12, 18-21, + amp), *ERBB2* (19-21, + amp), *ERBB4* (3, 4, 6-9, 15, 23), *ERCC2* (2, 3, 8-10, 15, 19-21), *ESR1* (6-10), *FGFR1* (4, 7, + amp), *FGFR2* (6, 8, 11, + amp), *FGFR3* (7, 9, 14, 16, 18, + amp), *FGFR4* (9, 12, 13, 16, + amp), *FLT1, FLT3* (11, 14, 16, 20), *FLT4, GLI2* (5, 13), *GNAS* (8, 9), *GSK3B* (2, 5, 10), *HRAS* (2, 3), *IDH1* (4), *IDH2* (4), *JAK2* (14), *JAK3* (4, 13, 16), *KDM6A, KDR* (6, 7, 11, 19, 21, 26, 27, 30), *KIT* (2, 9-11, 13-15, 17, 18), *KRAS* (2-4), *MAP2K1* (1-3, 6), *MAP2K2, MAP2K4, MAP3K1, MDM2* (3, 8, 11), *MET* (2, 11, 14, 16, 19), *MPL* (10), *MSTR1, NF1, NF2, NRAS* (2-4), *NRG1, PDGFRA* (12, 14, 15, 18), *PIK3CA* (2, 5, 7, 8, 10, 14, 19, 21), *PIK3R4* (2, 9, 18), *POLD1, POLE* (3-13), *PTCH1, PTEN, RAF1, RET* (10, 11, 13, 15, 16), *RHEB, ROS1* (35-40), *SMARCB1, SMO* (3, 5, 6, 8, 9, 11), *STK11, TP53* (2, 4-11), *VHL* (1-3).
Predictive Analysis for Therapy DNA gene panel version 2.0 (Radboud university medical centre)	Yes	Yes	Yes	Yes	Yes	No	No	No	Predictive Analysis for Therapy DNA gene panel version 2.0 (Radboud university medical centre)

Predictive Analysis for Therapy DNA gene panel version 3.0 (Radboud university medical centre)	Yes	Yes	Yes	Yes	Yes	Yes	Yes	No	Predictive Analysis for Therapy DNA gene panel version 3.0 (Radboud university medical centre)
SeqCap solid tumour 69 gene panel	Yes	Yes	Yes	Yes	Yes	Yes	Yes	No	*AKT1* (exons 3, 4, 11), *ALK* (19-29), *APC* (6, 7, 9, 12, 14, 16), *AR* (4, 5, 8), *BAP1* (1-17), *BRAF* (6, 7, 11, 12, 14, 15), *BRCA1* (2-23), *BRCA2* (2-27), *CCND1* (1-5), *CDK4* (2-8), *CDK6* (2-8), *CDKN2A* (1-3), *CDKN2B* (1, 2), *CTNNB1* (2, 3, 4, 7, 8), *DDR2* (4- 19), *DICER1* (26, 27), *DPYD* (14, int10-11, 11, 13, 22), *EGFR* (3, 7, 12, 15, 18, 19, 20, 21), *ERBB2* (8, 11-22, 27), *ERBB3* (1- 28), *ERBB4* (1-28), *ESR1* (4-8), *FBXW7* (4, 5, 7-12), FGFR1 (13, 15), *FGFR2* (7, 9, 12, 14), *FGFR3* (7, 9, 14, 16*)*, *FOXL2* (1), *FRK* (6), *GATA3* (2-6), *GNA11* (4, 5), *GNAQ* (4, 5), *GNAS* (7-9), *H3F3A* (2), *H3F3B* (2), *HIST1H3B* (1), *HIST1H3C* (1), *HNF1A* (1-9), *HRAS* (2-4), *IDH1* (4), *IDH2* (4), *IL6ST* (6, 10), *JAK1* (15, 16), *JAK2* (12, 14), *KIT* (2, 8-10, int10-11, 11, 13, 14, 17, 18), *KRAS (2-4)*, *MAP2K1* (1-11), *MET* (2-13, int13-14, 14, int14-15, 15- 21), *NRAS* (2-4), *NTRK1* (14, 15), *NTRK3* (16, 17), *PDGFRA* (12, 14, 18), *PIK3CA* (2, 3, 5, 8, 10, 14, 21), *PIK3R1* (2-16), *POLE* (9, 13, 14), *PTEN* (1-9), *RB1* (1-27), *RET* (1-20), *RNF43* (2-10), *ROS1* (37, 38, 41), *SMAD4* (2-12), *SMARCA4* (3-36), *SMARCB1* (1-9), *SMO* (1-12), *SPOP* (5, 6), *STAT3* (3, 6, 17, 20, 21), *STK11* (1-9), *TERT* (promotor), *TP53* (2-11), *VHL* (1-3).
TruSeq Amplicon Cancer Panel version 1.0	Yes	Yes	Yes	Yes	Yes	Yes	Yes	Yes	Mutation hotspots: *ABL1, AKT1, ALK, APC, ATM, BRAF, CDH1, CDKN2A, CSF1R, CTNNB1, EGFR, ERBB2, ERBB4, FBXW7, FGFR1, FGFR2, FGFR3, FLT3, GNA11, GNAQ, GNAS, HNF1A, HRAS, IDH1, JAK2, JAK3, KDR, KIT, KRAS, MET, MLH1, MPL, NOTCH1, NPM1, NRAS, PDGFRA, PIK3CA, PTEN, PTPN11, RB1, RET, SMAD4, SMARCB1, SMO, SRC, STK11, TP53, VHL*.
TruSight Oncology 500 actionable targets panel (Radboud university medical centre)	Yes	Yes	Yes	Yes	Yes	Yes	Yes	Yes	Mutation hotspots version 1: *ALK, AR, ATM, B2M, BARD1, BRAF, BRCA1, BRCA2, BRIP1, CCND1, CDK12, CDK4, CDK6, CDKN2A, EGFR, ERBB2, ERBB4, FANCL, FGFR1, FGFR2, FGFR3, FGFR4, HRAS, JAK1, JAK2, KIT, KRAS, MAP2K1, MAP2K2, MAP2K4, MAP3K1, MET, MLH1, MSH2, MSH6, NOTCH1, NOTCH2, NOTCH3, NOTCH4, NRAS, PALB2, PDGFRA, PDGFRB, PIK3CA, POLE, PPP2R2A, PTEN, RAD51B, RAD51C, RAD51D, RAD54L, RAF1, RET, TP53*.<br><br>In version 2, the following mutation hotspots were added: *AKT1, AKT2, AKT3, ATR, CCND2, CCND3, CHEK1, CHEK2, CSF1R, FANCA, FANCC, FANCD2, FANCF, FLT1, FLT3, FLT4, GNA11, GNAQ, GNAS, KDR, MRE11, MST1R, NBN, NF1, PIK3R1, PIK3R2, POLD1, PTCH1, RAD50, RAD51, TSC1, TSC2.*
Whole Exome Sequencing actionable targets panel version 2 (Radboud university medical centre)	Yes	Yes	Yes	Yes	Yes	Yes	Yes	Yes	Mutation hotspots: *AKT1, AKT2, AKT3, ALK, AR, ATM, ATR, B2M, BARD1, BRAF, BRCA1, BRCA2, BRIP1, CCND1, CCND2, CCND3, CDK12, CDK4, CDK6, CDKN2A, CHEK1, CHEK2, CSF1R, EGFR, ERBB2, ERBB4, FANCA, FANCC, FANCD2, FANCF, FANCL, FGFR1, FGFR2, FGFR3, FGFR4, FLT1, FLT3, FLT4, GNA11, GNAQ, GNAS, HRAS, JAK1, JAK2, KDR, KIT, KRAS, MAP2K1, MAP2K2, MAP2K4, MAP3K1, MET, MLH1, MRE11, MSH2, MSH6, MST1R, NBN, NF1, NOTCH1, NOTCH2, NOTCH3, NOTCH4, NRAS, PALB2, PDGFRA, PDGFRB, PIK3CA, PIK3R1, PIK3R2, POLD1, POLE, PPP2R2A, PTCH1, PTEN, RAD50, RAD51, RAD51B, RAD51C, RAD51D, RAD54L, RET, TP53, TSC1, TSC2*.

Abbreviations: CNA, copy number alteration; mut, mutation

Supplementary Table S2. Characteristics of patients with recurrent and/or metastatic disease.

	Total (n=464)	Adenoid cystic carcinoma (n=204)	Salivary duct carcinoma (n=181)	Acinic cell carcinoma (n=21)	Mucoepidermoid carcinoma (n=20)	Adenocarcinoma - not otherwise specified (n=12)	Myoepithelial carcinoma (n=9)	Polymorphous adenocarcinoma (n=6)	Epithelial-myoepithelial carcinoma (n=5)	Basal cell adenocarcinoma (n=3)	Secretory carcinoma (n=2)	Clear cell carcinoma (n=1)
**Age at diagnosis, median, years (range)**	59 (14-88)	53 (16-81)	65 (31-88)	53 (33-79)	56 (17-77)	66 (27-81)	49 (28-76)	54 (47-65)	63 (34-75)	44 (14-75)	42 (32-51)	68
**Sex, n (%)**												
Female	212 (46%)	124 (61%)	47 (26%)	8 (38%)	12 (60%)	5 (42%)	6 (67%)	4 (67%)	3 (60%)	2 (66%)	0	1 (100%)
Male	252 (54%)	80 (39%)	134 (74%)	13 (62%)	8 (40%)	7 (58%)	3 (33%)	2 (33%)	2 (40%)	1 (33%)	2 (100%)	0
**Primary tumour site, n (%)**												
Major salivary gland	304 (66%)	82 (40%)	167 (92%)	21 (100%	8 (40%)	10 (83%)	9 (100%)	0	3 (60%)	3 (100%)	1 (50%)	0
Parotid gland	230 (50%)	40 (20%)	140 (77%)	21 (100%)	8 (40%)	7 (58%)	7 (78%)	0	3 (60%)	3 (100%)	1 (50%)	0
Submandibular gland	63 (14%)	32 (16%)	26 (14%)	0	0	3 (25%)	2 (22%)	0	0	0	0	0
Sublingual gland	11 (2%)	10 (5%)	1 (1%)	0	0	0	0	0	0	0	0	0
Minor salivary gland	104 (22%)	80 (39%)	8 (4%)	0	5 (25%)	2 (17%)	0	6 (100%)	1 (20%)	0	1 (50%)	1 (100%)
Outside salivary glands	54 (112%)	41 (20%)	5 (3%)	0	7 (35%)	0	0	0	1 (20%)	0	0	0
Unknown	2 (0%)	1 (0%)	1 (1%)	0	0	0	0	0	0	0	0	0
**Initial treatment intent, n (%)**												
Curative	339 (73%)	168 (82%)	107 (59%)	17 (81%)	15 (75%)	9 (75%)	8 (89%)	5 (83%)	4 (80%)	3 (100%)	2 (100%)	1 (100%)
Palliative	125 (27%)	36 (18%)	74 (41%)	4 (19%)	5 (25%)	3 (25%)	1 (11%)	1 (17%)	1 (20%)	0	0	0
**Disease status at diagnosis of R/M disease, n (%)**												
Metastatic disease	309 (67%)	135 (66%)	131 (72%)	15 (71%)	9 (45%)	8 (67%)	5 (56%)	3 (50%)	2 (40%)	1 (33%)	0	0
Locoregional disease	100 (22%)	40 (20%)	36 (20%)	5 (24%)	6 (30%)	2 (17%)	3 (33%)	2 (33%)	2 (40%)	2 (66%)	1 (50%)	1 (100%)
Metastatic and locoregional disease	55 (12%)	29 (14%)	14 (8%)	1 (5%)	5 (25%)	2 (17%)	1 (11%)	1 (17%)	1 (20%)	0	1 (50%)	0
**Metastatic sites during disease course, n (%)**												
Bone	213 (46%)	75 (37%)	98 (54%)	11 (52%)	11 (55%)	5 (42%)	6 (67%)	3 (50%)	2 (40%)	1 (33%)	1 (50%)	0
Brain	77 (17%)	13 (6%)	50 (28%)	6 (29%)	3 (15%)	2 (17%)	0	0	2 (40%)	0	1 (50%)	0
Liver	112 (24%)	57 (28%)	47 (26%)	1 (5%)	1 (5%)	2 (17%)	2 (22%)	0	0	1 (33%)	1 (50%)	0
Lung	279 (60%)	151 (74%)	75 (41%)	17 (81%)	12 (60%)	4 (33%)	7 (78%)	3 (50%)	5 (100%)	2 (66%)	2 (100%)	1 (100%)
Lymph nodes	209 (45%)	55 (27%)	116 (64%)	9 (43%)	14 (70%)	6 (50%)	3 (33%)	3 (50%)	1 (20%)	1 (33%)	1 (50%)	0
Other	105 (23%)	23 (11%)	8 (4%)	5 (24%)	5 (25%)	3 (33%)	0	0	1 (20%)	0	0	0
**Time from primary diagnosis to R/M disease, median, months (range)**	16.1 (0.0–364.1)	32.0 (0.0–364.1)	9.5 (0.0–123.6)	16.7 (0.0–110.0)	10.3 (0.0–180.0)	5.3 (0.0–107.6)	17.8 (0.9–116.0)	42.5 (16.0–181.0)	10.1 (0.0–111.1)	26.5 (6.1–48.0)	6.9 (1.2–12.3)	49.0
**Palliative systemic therapy, n (%)**												
Yes	270 (58%)	77 (38%)	155 (86%)	10 (48%)	11 (55%)	5 (42%)	6 (66%)	2 (33%)	1 (20%)	0	2 (100%)	0
No	194 (42%)	127 (62%)	26 (14%)	11 (52%)	9 (45%)	7 (58%)	3 (33%)	4 (67%)	4 (80%)	3 (100%)	0	1 (100%)
**Lines of any palliative systemic therapy, including MMT, median (range)**	1 (0–7)	0 (0–4)	1 (0–7)	0 (0–2)	1 (0–7)	0 (0–3)	1 (0–3)	0 (0–1)	0 (0–1)	0 (0–0)	1 (1–1)	0

Abbreviations: MMT, molecular-matched therapy; R/M, recurrent and/or metastatic

Supplementary Table S3. Overview of applied next-generation sequencing panels and reasons for missing panels.

NGS Panel (total n=378)	N (%)
TruSight Oncology 500 actionable targets panel (Radboud university medical centre)	220 (58%)
Predictive Analysis for Therapy DNA gene panel version 2.0 (Radboud university medical centre)	51 (13%)
Whole Exome Sequencing actionable targets panel (Radboud university medical centre)	41 (11%)
Cancer Hotspot gene panel version 2.0 (Radboud university medical centre)	15 (4%)
Hartwig Medical Foundation gene panel	13 (3%)
Cancer Hotspot gene panel version 1.0 (Radboud university medical centre)	10 (3%)
Cancer Hotspot panel version 6 (Leiden University Medical Centre)	6 (2%)
Centre for Personalized Cancer Treatment gene panel	5 (1%)
Predictive Analysis for Therapy DNA gene panel version 3.0 (Radboud university medical centre)	3 (1%)
Oncopanel version 1 (Amsterdam University Medical Centre)	2 (1%)
General diagnostics panel version 5.1 (Erasmus Medical Centre)	1 (0.3%)
Cancer Hotspot Panel version 2Plus (University Medical Centre Utrecht)	1 (0.3%)
Cancer Hotspot Panel version 4 (Leiden University Medical Centre)	1 (0.3%)
EGFR and HER2 signal transduction gene panel version 2.0 and melanoma/GIST panel version 2.0 (Radboud university medical centre)	1 (0.3%)
Ion AmpliSeq Cancer Hotspot panel version 2	1 (0.3%)
Ion AmpliSeq panel Lung version 1	1 (0.3%)
Ion Torrent Oncomine focus DNA gene panel	1 (0.3%)
Maxima RNAseq gene panel version 24.1	1 (0.3%)
Oncomine Comprehensive Assay plus panel (Leiden University Medical Centre)	1 (0.3%)
SeqCap solid tumour 69 gene panel	1 (0.3%)
TruSeq Amplicon Cancer Panel version 1.0	1 (0.3%)

Reasons for missing NGS panels (total n=86)	N (%)
NGS unavailable at time of diagnosis	43 (50%)
Insufficient tumour material or DNA quality	13 (15%)
Patient unfit for systemic therapy due to poor performance status or comorbidity	11 (13%)
No indication for systemic therapy	9 (10%)
Patient refused biopsy	1 (1%)
Unclear	9 (10%)

Abbreviations: NGS, next-generation sequencing

Supplementary Table S4. Characteristics of salivary duct carcinoma patients treated with first-line androgen receptor axis-targeted therapy and with the first HER2-targeted therapy in any line.

	**First-line AR axis-targeted therapy (n=110)**	**First HER2-targeted therapy in any line (n=35)**
**Age, median, years (range)**	67 (37-88)	63 (36-82)
**Sex, n (%)**		
Female	26 (24%)	6 (17%)
Male	84 (76%)	29 (83%)
**Primary tumour site, n (%)**		
Major salivary gland	102 (93%)	32 (91%)
Parotid gland	85 (77%)	28 (80%)
Submandibular gland	17 (15%)	4 (11%)
Sublingual gland	0	0
Minor salivary gland	3 (3%)	2 (6%)
Outside salivary glands	4 (4%)	1 (3%)
Unknown	1 (1%)	0
**Androgen receptor status, percentage of cells with nuclear staining, n(%)**		
70-100%	95 (86%)	29 (83%)
40-69%	5 (5%)	2 (6%)
1-39%	8 (7%)	4 (11%)
Negative	2 (2%)	0
**HER2 status, n (%)**		
Positive[a]	21 (19%)	34 (97%)
IHC score 3+, ISH positive	12 (11%)	27 (77%)
IHC score 3+, ISH negative	1 (1%)	1 (3%)
IHC score 3+, ISH not determined	1 (1%)	0
IHC score 2+, ISH positive	1 (1%)	3 (9%)
IHC not determined, ISH positive	4 (4%)	3 (9%)
ERBB2 mutation, IHC score 2+ or 1+, ISH negative	2 (2%)	0
Low[b]	48 (44%)	1 (3%)
Negative	30 (27%)	0
Not determined	11 (10%)	0

Abbreviations: HER2, human epidermal growth factor receptor 2; IHC, immunohistochemistry; ISH, in situ hybridization

[a] Positive HER2 status was defined as positive IHC/ISH according to the ASCO/CAP guidelines [1], and/or if amplification or mutation(s) in the *ERBB2* gene were detected by NGS.

[b] Low HER2 status was defined as an immunohistochemical score of 1+ or 2+ and negative in situ hybridization.

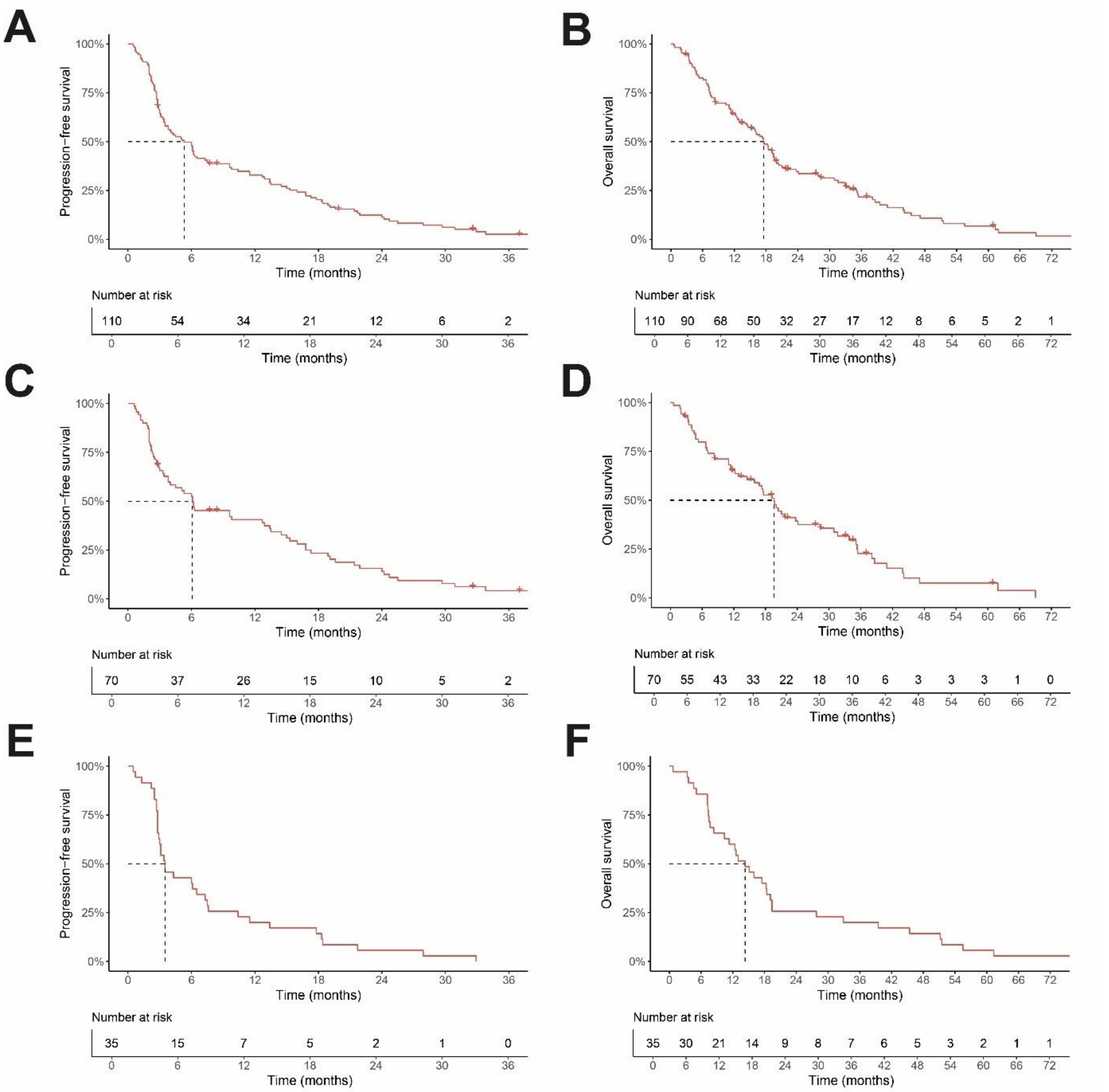

Supplementary Figure S1. Progression-free survival and overall survival with first-line androgen receptor (AR) axis-targeted therapy in patients with recurrent and/or metastatic salivary duct carcinoma. **A**, Progression-free survival with any AR axis-targeted therapy. **B**, Overall survival with any AR axis-targeted therapy. **C**, Progression-free survival with goserelin plus bicalutamide. **D**, Overall survival with goserelin plus bicalutamide. **E**, Progression-free survival with bicalutamide monotherapy. **F**, Overall survival with bicalutamide monotherapy.

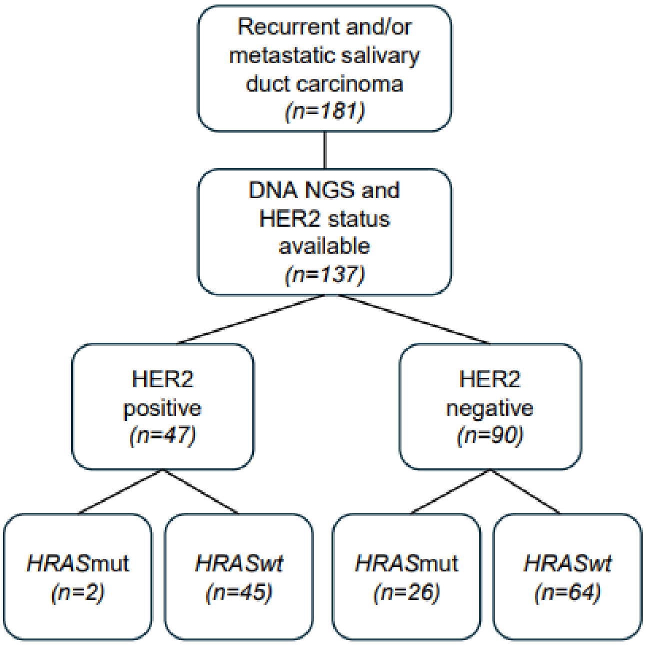

Supplementary Figure S2. Flow diagram of patients with recurrent and/or metastatic salivary duct carcinoma (n=181) who had available DNA next-generation sequencing and HER2 status (according to ASCO/CAP guidelines [24]). Patients are stratified by HER2 status (positive versus negative) and *HRAS* genotype (mutation versus wild type).Abbreviations: HER2, human epidermal growth factor receptor 2; mut, mutation; NGS, next-generation sequencing; wt, wild-type.

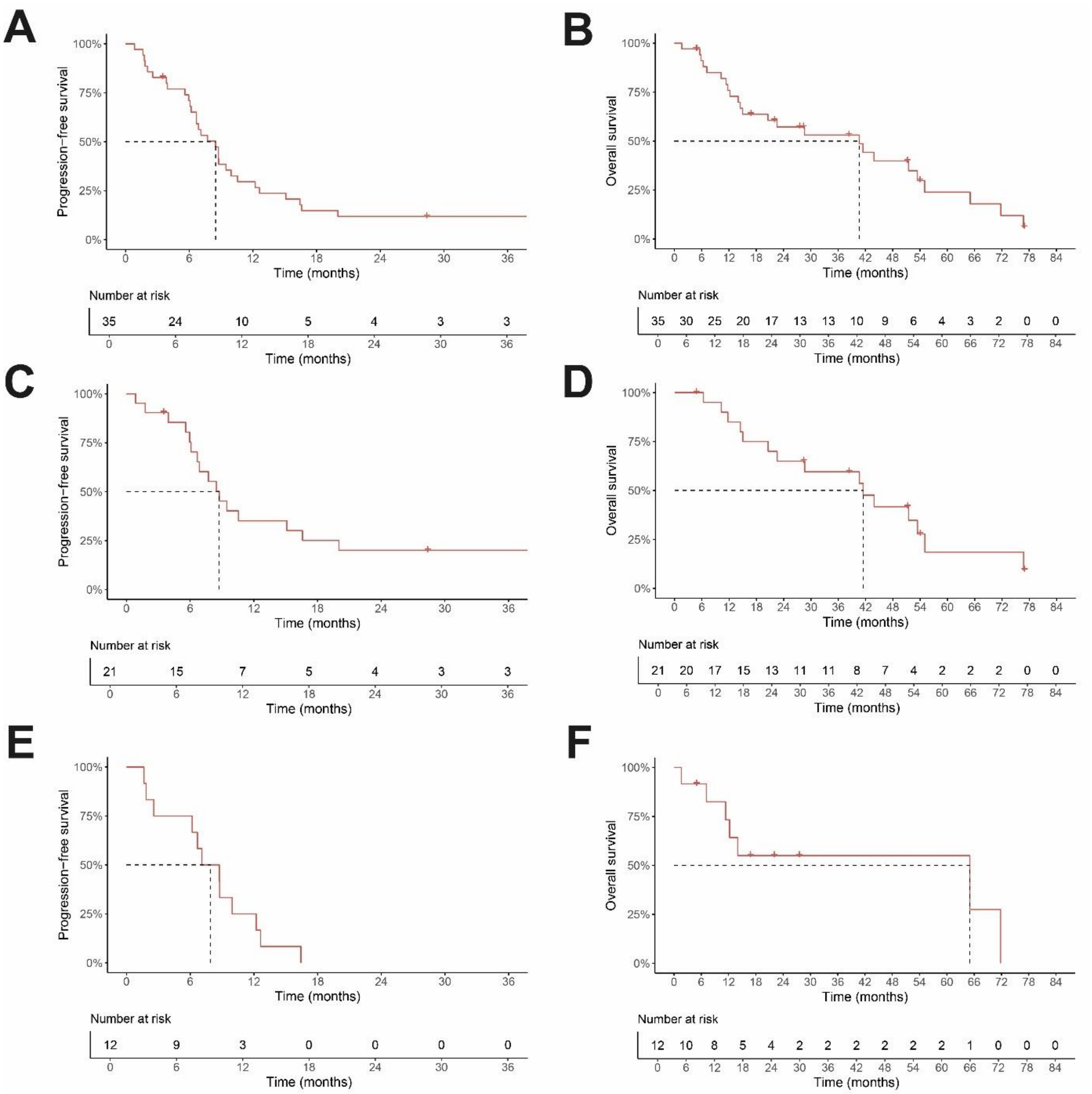

Supplementary Figure S3. Progression-free survival and overall survival with human epidermal growth factor receptor 2 (HER2)-targeted therapy in patients with recurrent and/or metastatic salivary duct carcinoma. **A**, Progression-free survival with the first HER2-targeted therapy, any regimen. **B**, Overall survival with the first HER2-targeted therapy, any regimen. **C**, Progression-free survival with pertuzumab/trastuzumab/docetaxel as the first HER2-targeted therapy. **D**, Overall survival with pertuzumab/trastuzumab/docetaxel as the first HER2-targeted therapy. **E**, Progression-free survival with trastuzumab/docetaxel (n=11) or trastuzumab/paclitaxel (n=1) as the first HER2-targeted therapy. **F**, Overall survival with trastuzumab/docetaxel (n=11) or trastuzumab/paclitaxel (n=1) as the first HER2-targeted therapy.

References

1 Wolff AC, Hammond MEH, Allison KH et al. Human Epidermal Growth Factor Receptor 2 Testing in Breast Cancer: American Society of Clinical Oncology/College of American Pathologists Clinical Practice Guideline Focused Update. J Clin Oncol 2018; 36 (20): 2105-2122.
